# An Interesting Case of Acute Transverse Myelitis Secondary to Hepatitis C and Tricuspid Valve Endocarditis: A Case Report

**DOI:** 10.7759/cureus.75312

**Published:** 2024-12-08

**Authors:** Vikram B Vikhe, Vivek H Lapsiwala, Ahsan A Faruqi, Tejas Kore, Ahanaa Chakraborty

**Affiliations:** 1 General Medicine, Dr. D. Y. Patil Medical College, Hospital and Research Centre, Dr. D. Y. Patil Vidyapeeth (Deemed to Be University), Pune, IND

**Keywords:** acute transverse myelitis, hepatitis c, infective endocarditis, methicillin-sensitive staphylococcus aureus, paraparesis, spinal cord inflammation, vegetations

## Abstract

Acute transverse myelitis (ATM) is a neurological disorder characterized by inflammation of the spinal cord, often resulting in sensory, motor, and autonomic dysfunction. Herein, we present a unique case of acute transverse myelitis secondary to hepatitis C virus (HCV) infection complicating infective endocarditis (IE), a rarely reported association. A 29-year-old female presented with progressive lower extremity weakness, urinary retention, and sensory disturbances. Clinical examination revealed bilateral lower limb weakness with diminished deep tendon reflexes and sensory loss below the T8 level. Magnetic resonance imaging (MRI) of the spine demonstrated T2 hyperintensity spanning multiple spinal segments consistent with transverse myelitis. Further investigations revealed the presence of HCV infection with evidence of active viremia. Additionally, echocardiography demonstrated vegetation on the tricuspid valve consistent with IE. The patient underwent a thorough infectious workup, which confirmed the diagnosis of IE as blood cultures showed growth of methicillin-sensitive Staphylococcus aureus (MSSA). This case highlights the importance of considering acute transverse myelitis as a potential neurological complication of HCV infection, particularly in the context of IE.

## Introduction

Acute transverse myelitis (ATM) is a rare focal inflammatory condition of the spinal cord usually presenting with rapid onset motor weakness and sensory and autonomic involvement [1]. The etiology of ATM can be diverse, including idiopathic origins, post-infectious complications, systemic inflammatory conditions, or multifocal central nervous system (CNS) diseases [1,2]. Transverse myelitis associated with hepatitis C infection has been previously reported [3,4]. Hepatitis C virus (HCV) is a single-stranded RNA virus belonging to the Flaviviridae family, known to cause chronic liver disease and a variety of extrahepatic manifestations, including neurological complications [5,6].

Infective endocarditis (IE), an inflammation of the endocardium and heart valves primarily caused by bacterial infections, presents diverse symptoms and complications [7,8]. Staphylococcus aureus is the most common causative agent of IE, often leading to severe systemic infections [7]. Major neurological complications of IE typically result from embolization from endocardial vegetation, leading to cerebral infarcts and hemorrhages. However, septicemia and embolization can also provoke an exaggerated immune response in the spinal cord, potentially resulting in transverse myelitis [9].

The initial treatment for ATM typically involves high-dose corticosteroids, which aim to reduce inflammation and immune response. Intravenous immunoglobulin (IVIG) is another option for patients who do not respond to corticosteroids. It involves administering immunoglobulins to modulate the immune response and reduce inflammation. IVIG at a dose of 2 g/kg over two to five days, administered in divided doses [1,10,11]. If ATM is secondary to an infectious process such as bacterial IE, broad-spectrum antibiotics are required. In general, a combination of Vancomycin as a principal agent and Ceftriaxone as a second agent is used as an empirical treatment for native valve endocarditis [7,8]. Sofosbuvir-Velpatasvir is a combination antiviral drug used to treat HCV infections. It uses sofosbuvir, a nucleotide polymerase inhibitor, and velpatasvir, an NS5A inhibitor, to target and suppress several phases of the HCV life cycle. This combination has been found to be efficacious across all genotypes of HCV and is generally well-tolerated with a favorable safety profile [12].

## Case presentation

A 29-year-old Indian female presented to the emergency department with bilateral lower limb weakness for two days, associated with a band-like sensation in the chest. She experienced tingling and numbness in her toes that progressed to her hips, accompanied by a loss of bowel and bladder sensation. The patient gave a history of fever with chills and rigor, intermittent in nature, relieved by medication for 14 days. She had undergone a blood transfusion 15 days prior for anemia (Hb <7 g/dL).

**Figure 1:**
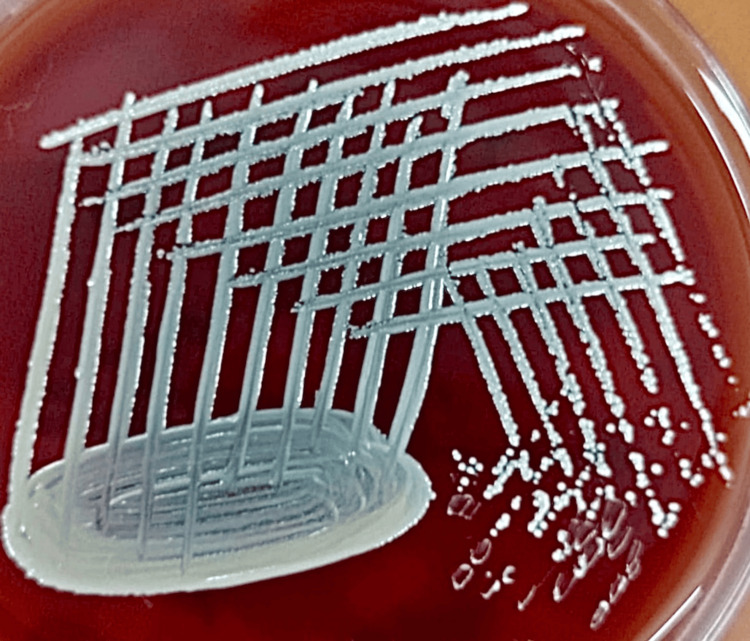
Blood agar showing growth of Staphylococcus aureus sensitive to methicillin

**Figure 2:**
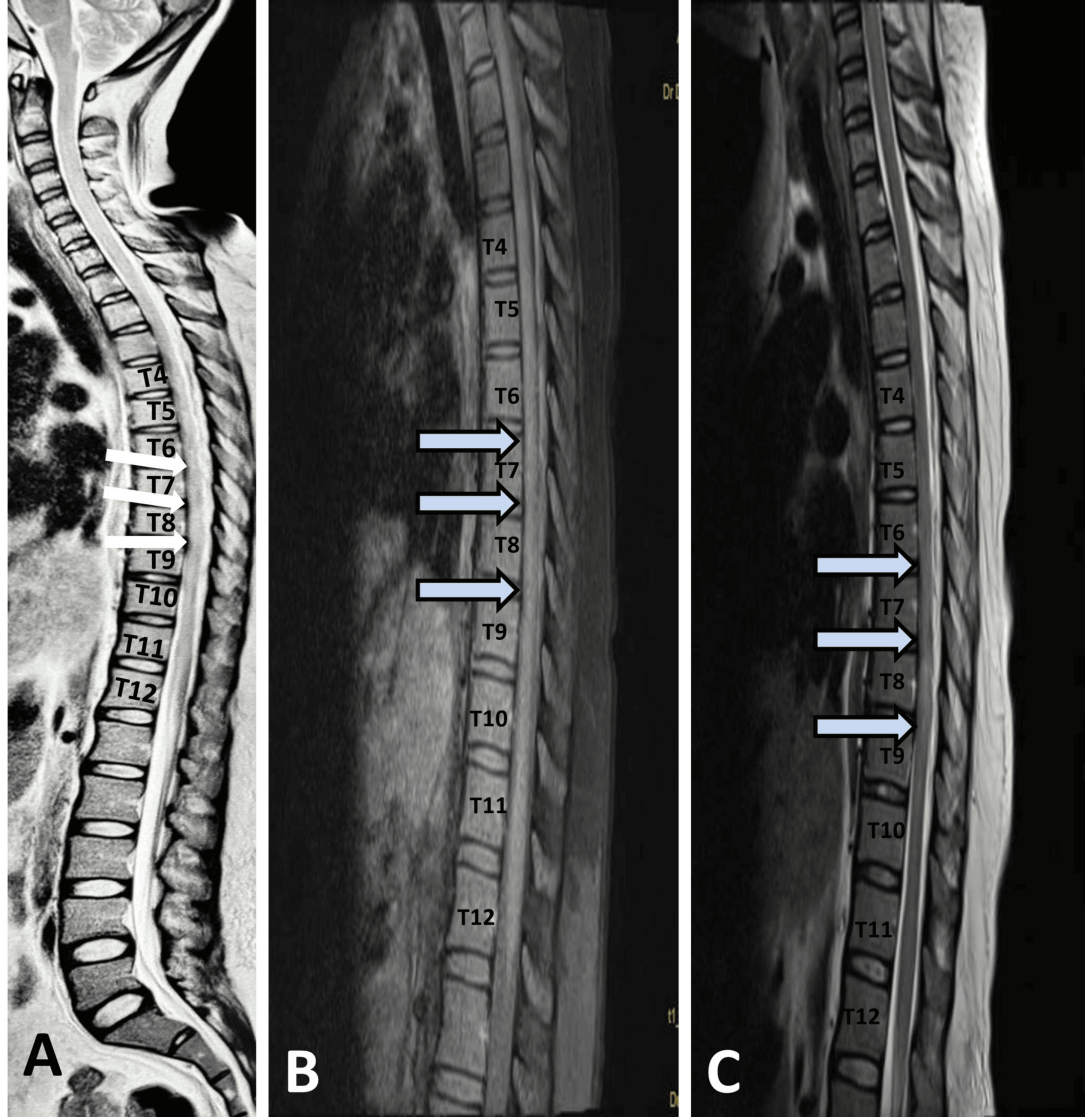
MRI whole spine screening with dedicated thoracic spinal cord (plain + contrast). The images demonstrate changes in the thoracic spinal cord, particularly from T6 to T9, which are suggestive of transverse myelitis. This is characterized by increased signal intensity on T2-weighted images and could be further clarified with contrast enhancement as seen in image C A: T1-weighted sagittal MRI image of the whole spine showing the hypointense signal from T6 to T9. B: T2-weighted sagittal MRI image of thoracic spinal cord showing increased signal intensity in the spinal cord from approximately T6 to T9, highlighted by the arrows. C: T2-weighted sagittal MRI image with contrast of thoracic spinal cord showing increased signal intensity in the spinal cord from T6 to T9. MRI, magnetic resonance imaging

On admission, her blood pressure was 130/80 mm Hg, pulse was 120 beats/minute, respiratory rate was 18 breaths/minute, and oxygen saturation was 98% on room air. On neurological examination, higher mental functions were preserved, and cranial nerves were intact. There was spasticity and hyperreflexia in both lower limbs, muscle power of 1/5 at the hip and knee, and 0/5 at the ankles, with extensor plantar reflexes bilaterally. Hypotonia was noted in bilateral lower limbs. Sensory examination showed reduced sensation to touch, pain, and temperature in both lower limbs. Superficial abdominal reflexes were absent, and beavor’s sign was absent localizing the sensory level at below T6. The anal tone was decreased. Abdominal examination revealed a distended abdomen with a palpable liver and urinary bladder. The patient had hesitancy of urine, and hence Foley’s catheterization was done. Routine investigations, along with blood and urine cultures, were sent. Day 1 reports (Table 1) indicated an HCV infection with a viral load of over 6.4 million/dL. Cerebrospinal fluid (CSF) analysis revealed lymphocytic pleocytosis with raised protein levels and normal glucose levels (Table 2). Blood culture showed growth of methicillin-sensitive Staphylococcus Aureus (MSSA), sensitive to Vancomycin (Figure 1). Magnetic resonance imaging (MRI) brain and spine with contrast revealed a hyperintense T2 signal in the dorsal spinal cord from T6-T9, indicating transverse myelitis (Figure 2). Transthoracic echocardiography revealed moderate tricuspid regurgitation, moderate pulmonary arterial hypertension, tricuspid vegetation seen as a hyperechogenic structure noted on the tricuspid leaflet measuring 1.27x1.32 cm, and an LVEF of 60%. Abdominal ultrasonography showed an enlarged liver (18.3 cm) with normal echotexture.

**Table 1:** Blood investigations done on day 1 of admission Hb, hemoglobin; TLC, total leucocyte count; SGOT, serum glutamic-oxaloacetic  transaminase; SGPT, serum glutamic-pyruvic transaminase; HCV, hepatitis C virus; CRP, C-reactive protein; ESR, erythrocyte sedimentation rate

Values	Observed value	Normal range
Hb	9.0	11.6-15.0 g/dL
TLC	11,400	4000-10000 /µL
Platelet count	176000	150000-410000 /µL
Serum urea	23	17-49 mg/dL
Serum creatinine	0.6	0.6-1.35 mg/dL
Total bilirubin	0.5	0.2-1.2 mg/dL
SGOT	16	8-48 IU/dL
SGPT	16	7-55 IU/dL
HIV	Non-reactive	Non-reactive
HCV	Reactive	Non-reactive
HBsAg	Non-reactive	Non-reactive
Dengue (NS1, IgG, IgM)	Negative	Negative
Rapid malaria test	Negative	Negative
Widal	Negative	Negative
CRP	44.90	0-10 mg/dL
ESR	68	0-20 mm/hour

**Table 2:** CSF analysis at the time of admission TLC, total leucocyte count; ADA, adenosine deaminase; CSF, cerebrospinal fluid

Values	Observed value	Normal range
Appearance	Clear, transparent	-
Quantity	2 mL	-
Cobweb	Absent	Absent
Proteins	72 mg/dL	15-45 mg/dL
Glucose	52 mg/dL	40-80 mg/dL
TLC	32/cu.mm	0-5 cells/cu.mm
Neutrophils	10%	-
Lymphocytes	90%	-
ADA	4	0-5 U/L

The patient received intravenous corticosteroids, antiviral therapy for HCV, and broad-spectrum antibiotics for IE. High-dose steroids (Methylprednisolone 1 g IV/day for five days) and empirical antibiotics (Vancomycin 1 g IV twice daily and Ceftriaxone 2 g IV daily) were started. Sofosbuvir-Velpatasvir (40 mg/10 mg) once daily was added for HCV infection. Despite initial treatment with steroids, the patient showed minimal neurological improvement, prompting the administration of IVIG 2 g/kg over five days, which led to better clinical outcomes. Antibiotics were continued for six weeks. Sofosbuvir-Velpatasvir was continued for a total of three months. Gradual improvement in neurological symptoms was observed over several weeks, including resolution of urinary retention and partial recovery of motor function. Follow-ups over several weeks were uneventful.

## Discussion

ATM is a rare but serious inflammatory condition of the spinal cord, leading to motor, sensory, and autonomic dysfunction. The etiology of ATM is diverse, including idiopathic origins, infections, autoimmune diseases, and paraneoplastic syndromes [1,2]. Recently, HCV has been increasingly recognized for its extrahepatic manifestations, particularly its role in neurological complications such as ATM. Several case reports have suggested that HCV-related immune responses play a significant role in the pathogenesis of ATM [3,5].

HCV is known to induce a hyperimmune state, which can result in vasculitis and demyelination within the CNS. The virus's ability to affect multiple organ systems highlights the importance of considering HCV in patients presenting with unexplained neurological symptoms [5,6]. Studies have demonstrated that chronic HCV infection can lead to immune-mediated neurological disorders, with ATM being one of the more severe manifestations [5].

IE is another condition known for its neurological complications, including stroke, abscesses, and myelitis. IE involves infection of the heart's endocardial surface, often affecting the valves and leading to systemic emboli that can reach the CNS. The neurological sequelae of IE are significant, with myelitis being a rare but documented outcome [9]. This case involves a patient diagnosed with ATM, with an MRI confirming T2 hyperintensity in the thoracic spinal cord, alongside concurrent diagnoses of HCV and IE. This scenario suggests a multifactorial etiology where both infectious and immune-mediated mechanisms are at play.

Management of such complex cases involves a comprehensive approach to treat the underlying infections while addressing the neurological manifestations. For HCV, antiviral therapy with agents such as sofosbuvir/velpatasvir has shown effectiveness in reducing systemic complications and improving patient outcomes. These antivirals have been proven safe and effective in large-scale studies and are a cornerstone in the management of HCV-related complications [12].

For IE, prolonged antibiotic therapy is essential, targeting the specific pathogens involved. In some cases, surgical intervention may be necessary to manage valvular damage or persistent infection [7,8]. The use of IVIG and other immunomodulatory therapies has shown promise in reducing inflammation and promoting recovery in ATM patients. Early diagnosis and initiation of appropriate therapy are crucial in improving outcomes and preventing long-term neurological deficits [1,10,11].

This case study illustrates the critical need for a broad differential diagnosis in patients with acute neurological symptoms. It highlights the importance of a holistic approach that considers both infectious and immune-mediated etiologies. Early and accurate diagnosis, coupled with targeted treatment strategies, can significantly improve patient outcomes in complex conditions like ATM with underlying HCV and IE. 

## Conclusions

This case report emphasizes the need for identifying ATM as a possible consequence of HCV infection, particularly in the context of IE. The patient's presentation, including bilateral lower limb weakness, sensory disturbances, and autonomic dysfunction, along with MRI findings, confirmed ATM. Subsequent investigations revealed HCV infection and MSSA-induced IE. A comprehensive treatment approach, including corticosteroids, antibiotics, antiviral therapy, and IVIG, led to significant clinical improvement. This case highlights the necessity of a multidisciplinary approach in managing complex, overlapping conditions and emphasizes the importance of early identification and treatment to improve patient outcomes and prevent complications. Clinicians should maintain a broad differential diagnosis for acute neurological deficits, especially in the presence of systemic infections, to ensure timely and effective intervention.

## References

[1] Frohman EM, Wingerchuk DM (2010). Clinical practice: transverse myelitis. N Engl J Med.

[2] Lim PA (2019). Transverse myelitis. Essent Phys Med Rehabil.

[3] De Carli DM, Pannebeker J, Pedro FL, Haygert CJ, Hertz E, Beck Mde O (2009). Transverse myelitis associated to HCV infection. Braz J Infect Dis.

[4] Suzuki K, Takao M, Katayama Y, Mihara B (2013). Acute myelitis associated with HCV infection. BMJ Case Rep.

[5] Monaco S, Ferrari S, Gajofatto A, Zanusso G, Mariotto S (2012). HCV-related nervous system disorders. Clin Dev Immunol.

[6] Morozov VA, Lagaye S (2018). Hepatitis C virus: morphogenesis, infection and therapy. World J Hepatol.

[7] Baddour LM, Wilson WR, Bayer AS (2015). Infective endocarditis in adults: diagnosis, antimicrobial therapy, and management of complications: a scientific statement for healthcare professionals from the American Heart Association. Circulation.

[8] McDonald EG, Aggrey G, Aslan AT (2023). Guidelines for diagnosis and management of infective endocarditis in adults: a wikiguidelines group consensus statement. JAMA Netw Open.

[9] Heiro M, Nikoskelainen J, Engblom E, Kotilainen E, Marttila R, Kotilainen P (2000). Neurologic manifestations of infective endocarditis: a 17-year experience in a teaching hospital in Finland. Arch Intern Med.

[10] Absoud M, Brex P, Ciccarelli O (2017). A multicentre randomiSed controlled TRial of IntraVEnous immunoglobulin compared with standard therapy for the treatment of transverse myelitis in adults and children (STRIVE). Health Technol Assess.

[11] Nikolić M, Lević Z (1976). Comparative analysis of the therapy of acute transverse myelitis. Paraplegia.

[12] Cheng PN, Mo LR, Chen CT (2022). Sofosbuvir/velpatasvir for hepatitis C virus infection: real-world effectiveness and safety from a nationwide registry in Taiwan. Infect Dis Ther.

